# Comparative Efficacy of Multiple Therapies for the Treatment of Patients With Subthreshold Depression: A Systematic Review and Network Meta-Analysis

**DOI:** 10.3389/fnbeh.2021.755547

**Published:** 2021-10-08

**Authors:** Xiumin Jiang, Yongxin Luo, Yiwen Chen, Jinglan Yan, Yucen Xia, Lin Yao, Xiaotong Wang, Su He, Feixue Wang, Taiyi Wang, Yongjun Chen

**Affiliations:** ^1^South China Research Center for Acupuncture and Moxibustion, Clinical Medical College of Acupuncture, Moxibustion and Rehabilitation, Guangzhou University of Chinese Medicine, Guangzhou, China; ^2^Department of Biostatistics and Preventive Medicine, School of Basic Medical Sciences, Guangzhou University of Chinese Medicine, Guangzhou, China; ^3^Research Institute of Acupuncture and Moxibustion, Shandong University of Traditional Chinese Medicine, Jinan, China; ^4^Guangdong-Hong Kong-Macao Greater Bay Area Center for Brain Science and Brain-Inspired Intelligence, Guangzhou, China

**Keywords:** subthreshold depression, multiple therapies, network meta-analysis, systematic review, Bayesian analysis

## Abstract

**Background:** Subthreshold depression (SD) is considered to be the precursor stage of major depression, which is correlated with functional impairment and increased suicide rate. Although there are multiple therapies for the treatment of SD, the comparison and efficacy of various methods has yet to be evaluated. This study aimed to evaluate the efficacy of different therapies by performing a Bayesian network meta-analysis.

**Methods:** We searched eight databases on April 3, 2021. Center for Epidemiologic Studies Depression Scale (CES-D), Beck Depression Inventory scale (BDI), the Patient Health Questionnaire-9 (PHQ-9), and the Kessler Screening Scale for Psychological Distress (K-6) were used as efficacy outcomes. This Bayesian network meta-analysis used a fixed-effects model.

**Findings:** Twenty-one randomized controlled trials involving 5,048 participants were included in this study. The results suggested that electroacupuncture (MD −12.00, 95% CrI −15.00, −10.00), conventional acupuncture plus wheat-grain moxibustion (MD −9.70, 95% CrI −14.00, −5.30), and the Chinese traditional peripateticism pill plus group counseling (MD −9.00, 95% CrI −11.00, −6.70) had better efficacy than the control group (CG) in improving CES-D. For BDI outcome, bright light therapy (MD −9.70, 95% CrI −13.00, −6.00), behavioral activation program (MD −5.70, 95% CrI −6.10, −5.40), and dim light therapy (MD −6.30, 95% CrI −10.00, −2.20) were better than the CG. Tai chi (MD −3.00, 95% CrI −4.00, −2.00) was better than CG for PHQ-9 outcomes. Telephone-based cognitive behavioral treatment (MD −2.50 95% CrI −2.70, −2.30) was better than the CG for K-6 scores.

**Conclusion:** Our results suggest that electroacupuncture or bright light therapy appear to be the better choices in the treatment of SD. This study provide new insights into clinical treatment selection and may aid the development of guidelines for the management of SD.

## Graphical Abstract

Graphical abstract of the network meta-analysis. Note: CES-D, Center for Epidemiologic Studies Depression Scale; BDI, Beck Depression Inventory Scale; PHQ-9, the nine-Item Patient Health Questionnaire; K6, Kessler Screening Scale for Psychological Distress; SUCRA, surface area under the cumulative ranking curves.

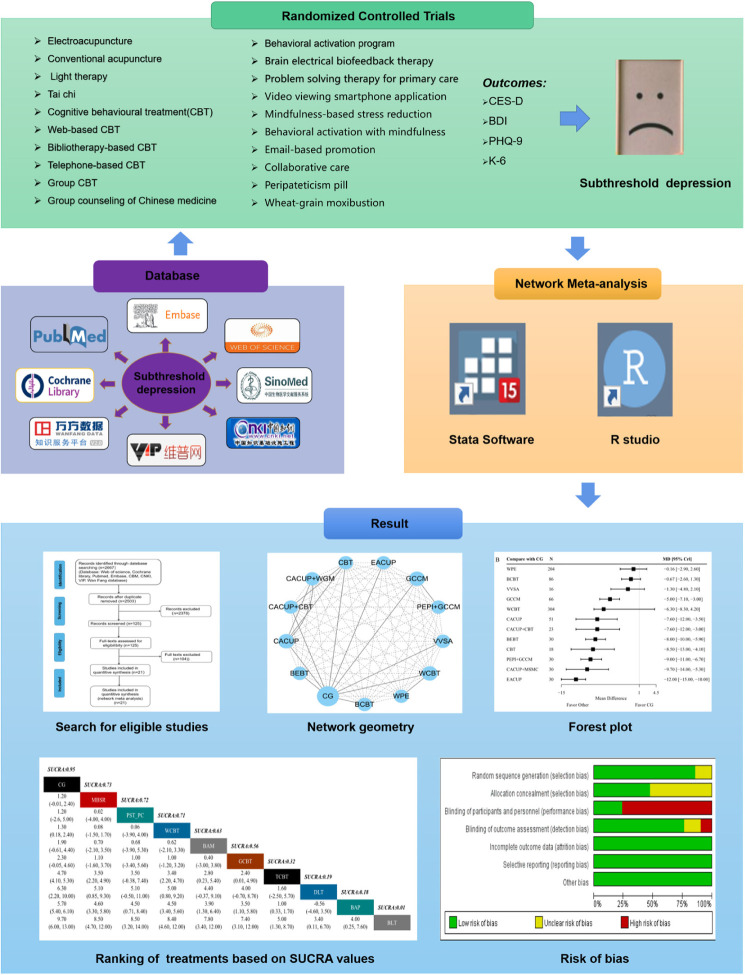

## Introduction

Subthreshold depression (SD) is defined in the Diagnostic and Statistical Manual of Mental Disorders as including “dysthymia,” “brief recurrent depression,” and “minor depressive disorder” (Keller et al., 1995), but not meeting criteria for major depressive disorder (Pincus et al., 1999). Population-based studies have found that SD has a wide prevalence, about 1.4–17.2% (Cuijpers and Smit, 2004), and the prevalence may be higher in the elderly or patients with chronic diseases (Cole and Dendukuri, 2003). Moreover, SD patients not only suffer from reduced quality of life, increased functional disability and mortality rate, but also require more service utilization and economic cost (Cuijpers and Smit, 2002; Cuijpers and Schoevers, 2004; Cuijpers et al., 2013). One third to one half of patients have moderate functional impairment, and at least 10–20% of patients have progressed to severe functional impairment at the 12-month follow-up (Jaffe et al., 1994; Kroenke, 2006; Lyness et al., 2006; Rodríguez et al., 2012). Studies have found that many patients with SD have persistent depressive symptoms, which is considered to be a risk factor for the development of major depressive disorder and other mental disorders (Cuijpers and Smit, 2004; Johnson et al., 2009). Thus, it is important to determine effective methods for the treatment of SD (Kroenke, 2017).

Early intervention may reduce the risk of symptom progression in patients with SD (Van’t Veer-Tazelaar et al., 2011). There are various treatment strategies for SD, including psychotherapy, pharmacotherapy, exercise therapy, and traditional Chinese medicine (TCM) therapy. Previous studies showed that different types of psychotherapy, including cognitive behavior therapy and behavior activation therapy, could reduce the Beck Depression Inventory scale (BDI) score of adults with SD and reduce the incidence of major depressive disorder over 12 months (Furukawa et al., 2012; Buntrock et al., 2015; He et al., 2019). In one meta-analysis of 700 patients, psychotherapy was shown to be more beneficial to patients with SD than care-as-usual approaches and it may prevent the onset of severe depression (Cuijpers et al., 2007). However, psychotherapy is often not readily accessible due to long-cycle and high cost of treatment (Juul et al., 2019). Pharmacotherapy for SD focuses on the use of antidepressants such as tricyclic drugs, 5-hydroxytryptamine, reuptake inhibitors, and others. Antidepressants are one of the main therapies used for moderate-to-severe depressive episodes, but one recent study found that antidepressants are often not better than the placebo for treating SD in randomized trials (Baumeister, 2012; Wang et al., 2019). Therefore, it still unclear whether antidepressants are suitable for the treatment of SD. Meanwhile, studies have confirmed that exercise, TCM psychotherapy, acupuncture, and moxibustion treatments are all effective in the treatment of SD, but there is still a lack of valid and reliable evaluation for these therapies (Tan et al., 2014; Gou et al., 2017; Li et al., 2017a; Xie, 2018). So far, no comparison of the treatment efficacy has been conducted among the above different therapies, which limits decision-making for patients with SD in the clinic and the future research for treatment of SD.

Network meta-analysis of clinical trials involves multivariate and multilevel analysis, which allows clinicians to assess and rank the efficacy of different treatments based on both direct and indirect comparisons (Higgins and Whitehead, 1996; Salanti et al., 2008; Shim et al., 2017). The statistical methods used in network meta-analysis are mainly divided into those with frequentist and Bayesian frameworks (Jansen et al., 2011). The advantages of Bayesian methods over frequentist methods are the use of informative priors and the possibility of hierarchical modeling, which may allow more comprehensive use of the information from historical and vertical data (Yin et al., 2017). Therefore, we conducted a Bayesian network meta-analysis in this study to identify the efficacy of multiple therapies for SD and rank the efficacy of different interventions. The result will help clinicians to choose the optimum prescription from multiple treatments for SD in practice.

## Materials and Methods

### Search Strategy

We searched the following databases: PubMed, Embase, Web of Science, Cochrane Central Register of Controlled Trials (CENTRAL), Chinese Biomedical Literature Database (CBM), China National Knowledge Infrastructure (CNKI), Wan Fang database, and the China Science and Technology Journal database (VIP) that were available by April 3, 2021, using these keywords: “subthreshold depression” OR “subsyndromic depression” AND “randomized” OR “random.”

### Inclusion and Exclusion Criteria

Studies were included according to the following criteria:

(1) Participants: patients included met the following criteria: they were diagnosed as SD based on the Diagnostic and Statistical Manual of Mental Disorders criteria (Sheehan et al., 1998) or Center for Epidemiologic Studies Depression Scale (CES-D) score ≥16 (Shima et al., 1984), BDI score ≥14, 7 $\underset{=}{<}$ 17-item Hamilton rating scale for depression score <17, or 8 $\underset{=}{<}$ 24-item Hamilton rating scale for depression score <20.

(2) Intervention: interventions in the treatment groups included acupuncture (electroacupuncture, conventional acupuncture), cognitive behavioral treatment (web-based cognitive behavioral treatment, bibliotherapy-based cognitive behavioral treatment, telephone-based cognitive behavioral therapy), video viewing smartphone application, behavioral activation with mindfulness, behavioral activation program, mindfulness-based stress reduction, problem solving therapy for primary care, email-based promotion, collaborative care, brain electrical biofeedback therapy, tai chi, bright light therapy, dim light therapy, group counseling with Chinese medicine, drug therapy (peripateticism pill), and moxibustion (wheat-grain moxibustion).

(3) Comparators: patients in the control group (CG) were the ones in a waiting list for treatment, under usual care, or having no treatments.

(4) Outcomes: outcomes in this study included at least one of the following evaluation indicators: CES-D, BDI, the 9-item patient health questionnaire (PHQ-9), or the Kessler screening scale for psychological distress (K-6).

(5) Types of studies that were included: randomized controlled trials (RCTs). The following studies were excluded: non-RCTs, single-arm design trials, conference abstracts, systematic reviews, or meta-analyses.

### Outcome Measures

We used CES-D as the primary outcome, with higher CES-D scores indicating more severe depressive symptoms. The secondary outcomes were BDI, PHQ-9, and K-6. The higher scores for BDI, PHQ-9 and K-6 indicate more severe depressive symptoms. The effect sizes for all outcomes were mean difference (MD) and 95% credible intervals (CrI).

### Data Collection and Quality Assessment

Two investigators independently scanned the titles and abstracts to confirm that the remaining studies met the predefined eligibility criteria, and full-text reviews were conducted for all potentially included studies. The data was extracted using a standard table, including information on the characteristics of the population, intervention(s), comparison(s), and outcome(s). The quality of each included trial was assessed by two authors based on the Cochrane Risk of Bias tool (Higgins et al., 2011).

### Statistical Analysis

We conducted a Bayesian framework network meta-analysis by using R studio 4.0. The research data included in this network meta-analysis were evaluated by the Markov chain Monte Carlo method with 10,000 burn-ins and 50,000 iterations of four each chain and a thinning interval of 10 (Ades et al., 2006). A Brooks Gelman Rubin diagnostic plot was used to assess model convergence and the potential scale reduction parameter (PSRF) was used to evaluate the convergence of the results. When 1.00 ≤ PSRF ≤ 1.05, it indicates that the results have good convergence and high reliability (Van Valkenhoef et al., 2012). I^2^ statistic and its 95% CrI were used to measure statistical heterogeneity, which is considered substantial when *I*^2^ > 50% (Higgins et al., 2003). Node-splitting analysis helps to determine the consistency test with an inconsistency model. In this study, a consistency model was chosen when the *p*-value of the node-splitting analysis was >0.05. If the *p*-value of the node-splitting analysis was <0.05, an inconsistency model was selected (Dias et al., 2010). We calculated the surface area under the cumulative ranking curves (SUCRA) to rank the curative effect of various interventions. The value range was 0–1. We used Begg’s test and Egger’s test to check publication bias, with a *p*-value of <0.05 indicating publication bias (Higgins et al., 2011). RevMan 5.3 software was used for bias risk assessment.

## Results

### Research Identification and Selection

**FIGURE 1:**
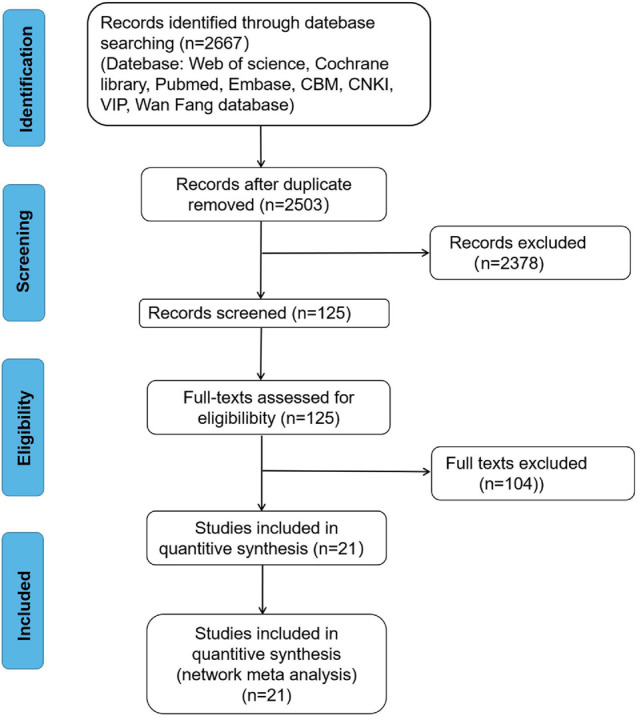
Preferred reporting items for systematic reviews and meta-analysis (PRISMA) flow diagram.

The literature search conducted retrieved 2,667 records. After deleting duplicates, 2,503 individual studies were recovered based on titles and abstracts. Among them, 125 studies were further selected for full-text review and 2,378 studies were removed due to the following reasons: non-SD, case report, animal models, republication, or review. Further examination excluded 104 studies according to the following reasons:

(1) The outcome was not the CES-D, BDI, PHQ-9, or K-6. (2) The studies were not RCTs. The remaining 21 studies were used for meta-analysis, including 5,048 patients (Figure 1). The characteristics of the selected trials are shown in Table 1.

**TABLE 1:** Characteristic of included studies.

**Study ID**	**Samples size**	**Scanning of SD**	**Treatment group interventions**	**Control group interventions**	**Course of treatment**	**Outcome**
Tan, 2020	60	CES-D ≥ 16; 7 ≤ HAMD-17 < 17	CACUP + WGM	CACUP	4 weeks	CES-D
Xie, 2018	60	CES-D ≥ 16; 7 ≤ HAMD-17 < 17	EACUP	CG	6 weeks	CES-D
Li et al., 2017b	60	ZYYXH/T49-2008	PEPI + GCCM	GCCM	12 weeks	CES-D
Zhang, 2015	79	CES-D ≥ 16; 7 ≤ HAMD-17 < 17	CACUP/CBT/CACUP + CBT/CG		8 weeks/6 weeks	CES-D
Dong et al., 2015	60	CES-D > 16	BEBT	CG	3 weeks	CES-D
Tan et al., 2014	72	7 ≤ HAMD-17 < 17	GCCM	CG	8 weeks	CES-D
Ebert et al., 2018	204	CES-D ≥ 16	WCBT	CG	12 weeks	CES-D
Buntrock et al., 2015	406	CES-D ≥ 16	WCBT	WPE	3–6 weeks	CES-D
Joling et al., 2011	170	CES-D ≥ 16	BCBT	CG	12 weeks	CES-D
Kageyama et al., 2021	32	CES-D ≥ 16	VVSA	CG	8 weeks	CES-D, K-6
Jiang et al., 2020	142	CES-D ≥ 16; BDI-II ≥ 14	BLT/DLT/CG		8 weeks	BDI-II
Spek et al., 2007	301	CES-D ≥ 12	WCBT/GCBT/CG		10 weeks	BDI-II
Furukawa et al., 2012	118	BDI-II ≥ 10; K-6 ≥ 9	TCBT	CG	8 weeks	BDI-II, K-6, PHQ-9
Kasckow et al., 2014	23	CES-D ≥ 11	PST-PC	CG	6–8 weeks	BDI-II
Zhang et al., 2019	56	BDI > 14; SDS > 53	MBSR	CG	8 weeks	BDI-II
Takagaki et al., 2018	118	BDI-II ≥ 10	BAP	CG	5 weeks	BDI-II
Imamura et al., 2014	762	NM	WCBT	CG	6 weeks	BDI-II, K-6
Wong et al., 2018	231	5 < PHQ-9 < 9	BAM	CG	8 weeks	BDI-II
Xie, 2020	63	CES-D ≥ 16	Tai Chi	CG	12 weeks	PHQ-9
Morgan et al., 2012	1326	5 < PHQ-9 < 9	EBP	CG	6 weeks	PHQ-9
Gilbody et al., 2017	705	DSM-IV	COC	CG	8 weeks	PHQ-9

*SD, subthreshold depression; CES-D, Center for Epidemiologic Studies Depression Scale; HAMD, Hamilton Rating Scale for Depression; BDI, Beck Depression Inventory Scale; K-6, Kessler Screening Scale for Psychological Distress; SDS, Self-rating Depression Scale; PHQ-9, the 9-item Patient Health Questionnaire; DSM-IV, Diagnostic and Statistical Manual of Mental Disorders (Fourth Edition); ZYYXH/T49-2008, Chinese society of traditional Chinese medicine issued the industry standard of depression; CACUP, conventional acupuncture; WGM, wheat-grain moxibustion; CG, control group; EACUP, electroacupuncture; PEPI, peripateticism pill; GCCM, group counseling with Chinese medicine; CBT, cognitive behavioral treatment; BEBT, brain electrical biofeedback therapy; WCBT, web-based cognitive behavioral treatment; BCBT, bibliotherapy-based cognitive behavioral treatment; VVSA, video viewing smartphone application; BLT, bright light therapy; DLT, dim light therapy; GCBT, group cognitive behavioral treatment; TCBT, telephone-based cognitive behavioral treatment; PST-PC, problem solving therapy for primary care; MBSR, mindfulness-based stress reduction; BAP, behavioral activation program; BAM, behavioral activation with mindfulness; EBP, email-based promotion; COC, collaborative care; WPE, web-based psycho education; NM, not mentioned.*

### Research Quality

We used the Cochrane Risk of Bias Tool to assess the quality of the above selected 21 studies. As shown in Supplementary Figure 1, 18 studies (85%) adopted a random sequence generation process using a computer random number generator or a random number table, 10 (47%) described the use of allocation or concealment methods, 5 (23%) described the blinding methods for researchers and participants, 16 (76%) described the blinding methods of outcomes assessment. In terms of incomplete outcome data, selective reporting and other bias, all included studies are assessed as low risk.

### Primary Outcome: Center for Epidemiologic Studies Depression Scale

**FIGURE 2:**
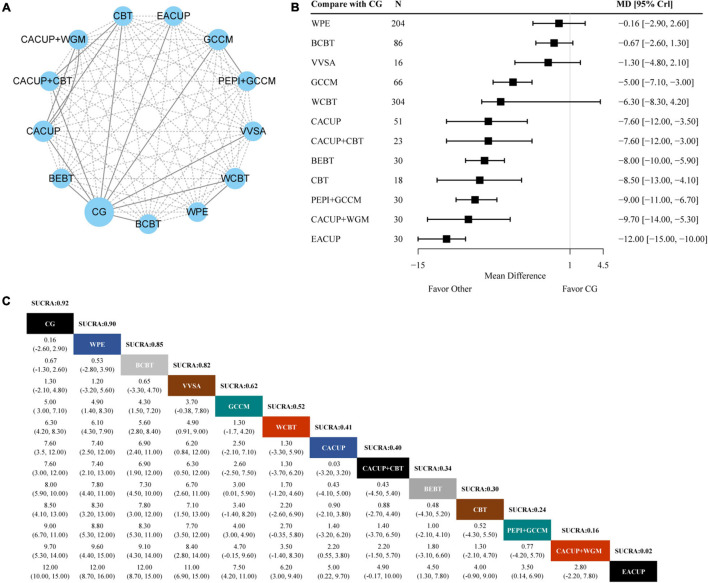
Results of the network meta-analysis for CES-D. **(A)** Network geometry of eligible comparisons for CES-D. **(B)** Forest plot of the network meta-analysis compared with control group. **(C)** Ranking of each intervention based on the SUCRA values and the league table for the relative effects of all treatments. EACUP, electroacupuncture; CACUP, conventional acupuncture; WGM, wheat-grain moxibustion; GCCM, group counseling with Chinese medicine; CBT, cognitive behavioral treatment; BEBT, brain electrical biofeedback therapy; WCBT, web-based cognitive behavioral treatment; VVSA, video viewing smartphone application; BCBT, bibliotherapy-based cognitive behavioral treatment; WPE, web-based psycho education; CG, control group; SUCRA, surface under the cumulative ranking curve; MD, weighted mean difference; CrI, credible interval.

In the network meta-analysis for the CES-D, 10 trials and 13 interventions were included, and the network plot is shown in Figure 2A. A forest plot showing the result of CG compared with other interventions is presented in Figure 2B. We found that most of the treatments show better efficacy on CES-D compared with CG alone. These interventions include electroacupuncture (MD −12.00, 95% CrI −15.00, −10.00), conventional acupuncture plus wheat-grain moxibustion (MD −9.70, 95% CrI −14.00, −5.30), the peripateticism pill plus group counseling with Chinese medicine (MD −9.00, 95% CrI −11.00, −6.70), cognitive behavioral treatment (MD −8.50, 95% CrI −13.00, −4.10), brain electrical biofeedback therapy instrument (MD −8.00, 95% CrI −10.00, −5.90), conventional acupuncture plus cognitive behavioral treatment (MD −7.60, 95% CrI −12.00, −3.00), conventional acupuncture (MD −7.60, 95% CrI −12.00, −3.50), group counseling with Chinese medicine (MD −5.00, 95% CrI −7.10, −3.00). SUCRA values is used to rank the curative effect of various treatments. As shown in Figure 2C, the results showed that electroacupuncture (2%) had the lowest SUCRA value across the various interventions, which means it had the highest probability of being the most effective treatment for CES-D. The following ranked treatments were conventional acupuncture plus wheat-grain moxibustion (16%), the peripateticism pill plus group counseling with Chinese medicine (24%), cognitive behavioral treatment (30%), brain electrical biofeedback therapy instrument (34%), conventional acupuncture plus cognitive behavioral treatment (40%), conventional acupuncture (41%), web-based cognitive behavioral treatment (52%), group counseling with Chinese medicine (62%), video viewing smartphone application (82%), bibliotherapy-based cognitive behavioral treatment (85%), and web-based psycho-education (90%).

### Secondary Outcome: Beck Depression Inventory Scale

**FIGURE 3:**
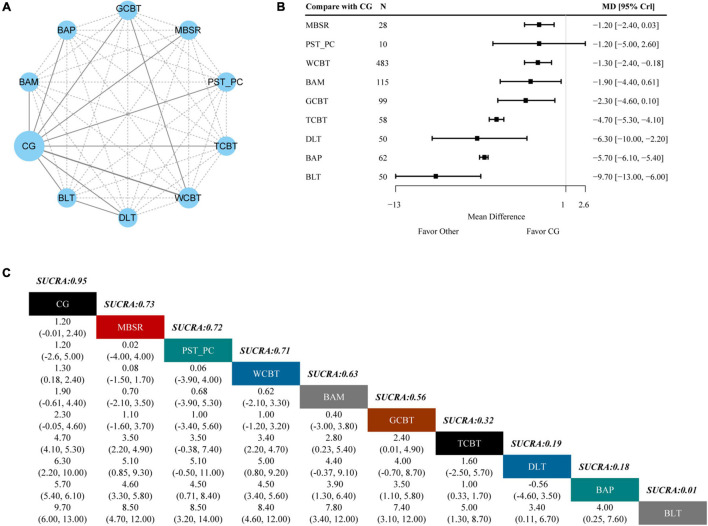
Results of the network meta-analysis for BDI. **(A)** Network geometry of eligible comparisons for BDI. **(B)** Forest plot of the network meta-analysis compared with control group. **(C)** Ranking of each intervention based on the SUCRA values and the league table for the relative effects of all treatments. BLT, bright light therapy; DLT, dim light therapy; BAP, behavioral activation program; TCBT, telephone-based cognitive behavioral treatment; GCBT, group cognitive behavioral treatment; BAM, behavioral activation with mindfulness; WCBT, web-based cognitive behavioral treatment; PST-PC, problem solving therapy for primary care; MBSR, mindfulness-based stress reduction; CG, control group; SUCRA, surface under the cumulative ranking curve; MD, weighted mean difference; CrI, credible interval.

Eight trials and ten interventions were involved in measuring BDI outcome. The network geometry is presented in Figure 3A. From the forest plot of Figure 3B, we found that bright light therapy (MD −9.70, 95% CrI −13.00, −6.00), behavioral activation program (MD −5.70, 95% CrI −6.10, −5.40), dim light therapy (MD −6.30, 95% CrI −10.00, −2.20), telephone-based cognitive behavioral treatment (MD −4.70, 95% CrI −5.30, −4.10), group cognitive behavior treatment (MD −2.30, 95% CrI −4.60, 0.10), behavioral activation with mindfulness (MD −1.90, 95% CrI −4.40, 0.61), web-based cognitive behavioral treatment (MD −1.30, 95% CrI −2.40, −0.18), and mindfulness-based stress reduction (MD −1.20, 95% CrI −2.40, 0.03) had better efficacy compared with CG alone on BDI. As shown in Figure 3C, Bright light therapy (1%) showed the lowest SUCRA value in all treatments, which indicates that it had the highest probability of being the most effective treatment for BDI for SD. The following ranked treatments were behavioral activation program (18%), dim light therapy (19%), telephone-based cognitive behavioral treatment (32%), group cognitive behavior treatment (56%), behavioral activation with mindfulness (63%), web-based cognitive behavioral treatment (71%), problem solving therapy for primary care (72%), and mindfulness-based stress reduction (73%).

### Secondary Outcome: Patient Health Questionnaire-9

**FIGURE 4:**
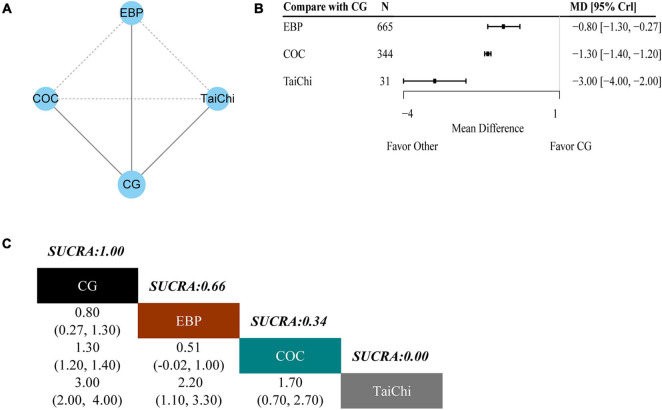
Results of the network meta-analysis for PHQ-9. **(A)** Network geometry of eligible comparisons for PHQ-9. **(B)** Forest plot of the network meta-analysis compared with control group. **(C)** Ranking of each intervention based on the SUCRA values and the league table for the relative effects of all treatments. EBP, email-based promotion; COC, collaborative care; CG, control group; SUCRA, surface under the cumulative ranking curve; MD, weighted mean difference; CrI, credible interval.

To evaluate the PHQ-9 outcome, three studies and four interventions were included. The network plot is shown in Figure 4A. As shown in the forest plot of PHQ-9 (Figure 4B), tai chi (MD −3.00, 95% CrI −4.00, −2.00), collaborative care (MD −1.30, 95% CrI −1.40, −1.20), and email-based promotion (MD −0.80 95% CrI −1.30, −0.27) showed better efficacy in PHQ-9 compared with CG alone. As shown in Figure 4C, tai chi (0%) had the lowest SUCRA value of the four interventions, which indicated that it had the highest probability of being the most effective treatment for PHQ-9 for SD. Other following effective treatments were collaborative care (34%) and email-based promotion (66%).

### Secondary Outcome: Kessler Screening Scale for Psychological Distress

**FIGURE 5:**
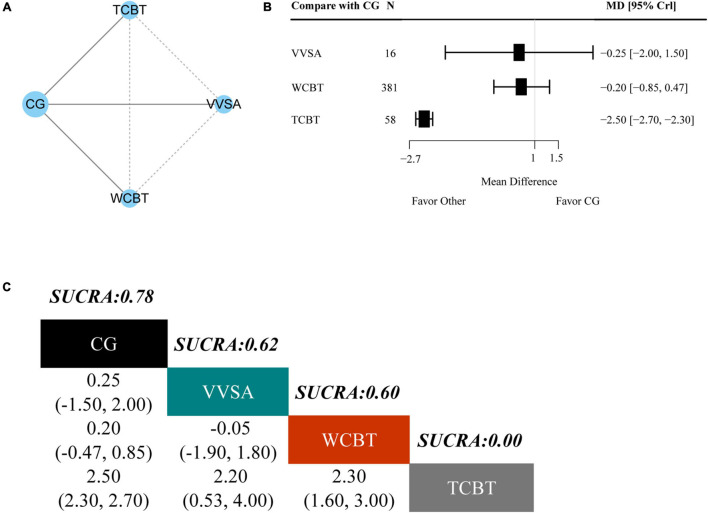
Results of the network meta-analysis for K-6. **(A)** Network geometry of eligible comparisons for K-6. **(B)** Forest plot of the network meta-analysis compared with control group. **(C)** Ranking of each intervention based on the SUCRA values and the league table for the relative effects of all treatments. TCBT, telephone-based cognitive behavioral treatment; WCBT, web-based cognitive behavioral treatment; VVSA, video viewing smartphone application; CG, control group; SUCRA, surface under the cumulative ranking curve; MD, weighted mean difference; CrI, credible interval.

There were three trials and four interventions that measured a K-6 outcome. The network plot is presented in Figure 5A. As indicated in the forest plot for K-6 (Figure 5B), telephone-based cognitive behavioral treatment (MD −2.50 95% CrI −2.70, −2.30) had better efficacy compared with CG alone. As shown in Figure 5C, telephone-based cognitive behavioral treatment (0%) had the lowest SUCRA values among the four interventions, which indicated that it had the highest probability of being the most effective treatment for K-6. The outcome from other two treatments were web-based cognitive behavioral treatment (60%) and video viewing smartphone application (62%), respectively.

### Model Convergence, Heterogeneity, and Publication Bias

The shrinking factors in the Brooks Gelman Rubin diagnostic plots for all outcomes were less than 1.05 (Supplementary Figure 2). The statistical heterogeneity for all outcomes were low (*I*^2^ < 50%, ranging from 5% to 17%). According to results of Begg’s and Egger’s tests with a funnel plot, no publication bias was detected (*p* > 0.05) in our study (Supplementary Figure 3 and Supplementary Table 1). There was no obvious inconsistency shown in this network meta-analysis.

## Discussion

In this network meta-analysis, our results provided evidence that all the involved treatments for patients with SD showed advantages over waiting for treatment or no treatment. Furthermore, we found that electroacupuncture was the best therapy to improve outcome on the CES-D. Meanwhile, bright light therapy was the optimum treatment for the outcome on the BDI and tai chi was best effective in promoting outcome on the PHQ-9. However, telephone-based cognitive behavioral treatment was the best intervention for outcome on the K-6.

Electroacupuncture has been widely used to treat psychiatric conditions including depression (Woo et al., 2015), which may be associated with multiple mechanisms. First, relevant research found that the serotonin system plays an important role in the pathophysiology of depression (Mouri et al., 2016). Electroacupuncture was reported to restore hippocampal CA1 synaptic plasticity by regulating the level of serotonin system receptors, thereby improving depression-like behavior (Han et al., 2018). Second, it has been found that proinflammatory cytokines play an important role in neurogenesis and neuroprotection (Kim et al., 2016). Patients with depression have high levels of pro-inflammatory cytokines, acute phase proteins, chemokines, and cell adhesion molecules (Raison et al., 2006). Electroacupuncture reduced hippocampal neuroinflammation in depressed rats by reducing the expression of pyrin domain-containing protein 3 inflammatory components (ASC and caspase-1), activating microglia and ATP gated transmembrane protein (P2 × 7) receptor as well as reducing levels of interleukin-1 beta (IL-1β), IL-18, IL-6, and tumor necrosis factor alpha (Yue et al., 2018; Li et al., 2021). Furthermore, hypothalamic pituitary adrenal axis dysregulation, which is generally considered a diagnostic criterion for early stages of depression, is also implicated in the pathology of depression (Du and Pang, 2015). Studies have shown that electroacupuncture could exert anti-depressive activity by regulating the hypothalamic pituitary adrenal axis (Le et al., 2016).

Consistent with our results, light therapy has been widely used to treat seasonal depression (Avissar et al., 1999). Light can affect mood and learning through different retinal brain pathways (Fernandez et al., 2018). The antidepressant effects of light therapy have been found to be related to changes in neurotransmitters, including serotonin and catecholamine (Lam et al., 1996; Neumeister, 2003). Previous studies also found that light therapy could synchronize the biological clock with the circadian rhythm of the environment, which is considered to be closely related to depression (Lam and Levitan, 2000; Pail et al., 2011; Jagannath et al., 2013). Furthermore, light therapy has the advantages of low cost, high security, and direct availability (Bais et al., 2016). Together, all these studies provide further evidence that light therapy may be the optimal treatment for patients with SD.

As an adjuvant therapy for depression, physical exercise has attracted increasing attention (Legrand and Neff, 2016). One study found that aerobic exercise can increase the levels of brain-derived neurotrophic factor, which has been shown to decrease in individuals with severe depression (Bocchio-Chiavetto et al., 2010). Tai chi was reported to ameliorate the depressive symptoms of elderly Chinese patients (Cho, 2008). Meanwhile, other studies provide evidences that tai chi could regulate brain networks associated with depression and alleviate depressive symptoms by regulating cortisol levels and immune system (Walther et al., 2018; Kong et al., 2019). However, given that the sample sizes of clinical research studies were small, whether tai chi has a unique benefit for treatment of SD over other physical exercise requires further study.

Our results suggested that telephone-based cognitive behavioral treatment is the best treatment for improving K-6, which is used to screen for mental health problems and measure the severity of the impact of these problems (Mitchell and Beals, 2011). Telephone-based cognitive behavioral treatment is a novel method for the treatment of SD (Furukawa et al., 2012). It may overcome some of the obstacles preventing patients from receiving traditional psychotherapy services, including occupational or social constraints, residency in underserved areas, and the need to commute, and therefore may be a more appropriate choice in these situations (Bee et al., 2008). Taken together, our findings suggest that more attention should be paid to the application of telephone-based cognitive behavioral treatment.

There were some limitations in our study. First, many RCT studies did not include PHQ-9 and K-6, which limited the comprehensive evaluation of the efficacy on the two outcomes. Second, parts of the quality assessment risk were unsatisfactory because of the characteristics of waiting for treatment or no treatment, it hard to blind both therapist and participants. Finally, given that the number of patients included was relatively small, more multi-center and high-quality RCTs are needed in the future to validate our findings.

## Conclusion

The results of this comprehensive network meta-analysis provides a complete evaluation of currently available therapies for patients with SD. Our results suggest that electroacupuncture or bright light therapy may be the preferred selection in the treatment of SD. This study provide new insights into clinical treatment selection and may help the development of guidelines for the management of SD.

## Data Availability Statement

The original contributions presented in the study are included in the article/Supplementary Material, further inquiries can be directed to the corresponding author.

## Author Contributions

YJC and XJ designed this network meta-analysis. XJ, YL, YWC, JY, and SH collected the data. XJ, YX, XW, YL, and YWC analyzed the data. XJ, LY, FW, and TW performed the analysis. XJ and YJC wrote the manuscript. All authors contributed to writing of this manuscript and approved the final version of the manuscript.

## Conflict of Interest

The authors declare that the research was conducted in the absence of any commercial or financial relationships that could be construed as a potential conflict of interest.

## Publisher’s Note

All claims expressed in this article are solely those of the authors and do not necessarily represent those of their affiliated organizations, or those of the publisher, the editors and the reviewers. Any product that may be evaluated in this article, or claim that may be made by its manufacturer, is not guaranteed or endorsed by the publisher.
