# Supplementary material for: Comparative Efficacy of Multiple Therapies for the Treatment of Patients With Subthreshold Depression: A Systematic Review and Network Meta-Analysis
Source: Front Behav Neurosci. 2021 Oct 8;15:755547. doi: 10.3389/fnbeh.2021.755547 (PMC8531252; doi:10.3389/fnbeh.2021.755547)
Supplement: Supplementary file 1 [file Data_Sheet_1.PDF]

Supplementary Material

1. Supplementary figure

Supplementary Figure 1. The risk of bias assessment graph of individual RCTs.

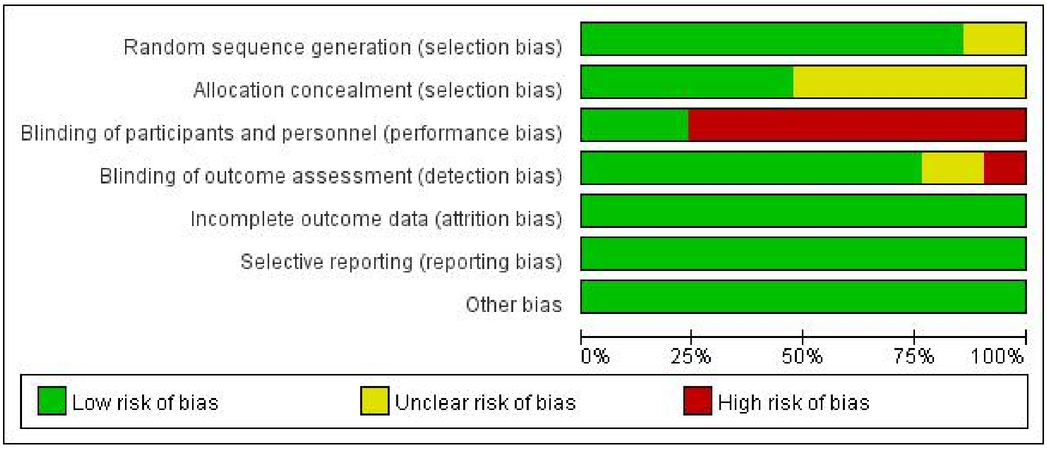

Supplementary Figure 2. Brooks Gelman Rubin diagnostic plots for model convergence with 10000 burn-ins and 50000 iterations of 4 each chain. (A) CES-D; (B) BDI; (C) PHQ-9; (D) K-6. Abbreviations: CES-D, Center for Epidemiologic Studies Depression scale; BDI, Beck Depression Inventory Scale; PHQ-9, the 9-item Patient Health Questionnaire; K6, Kessler Screening Scale for Psychological Distress.

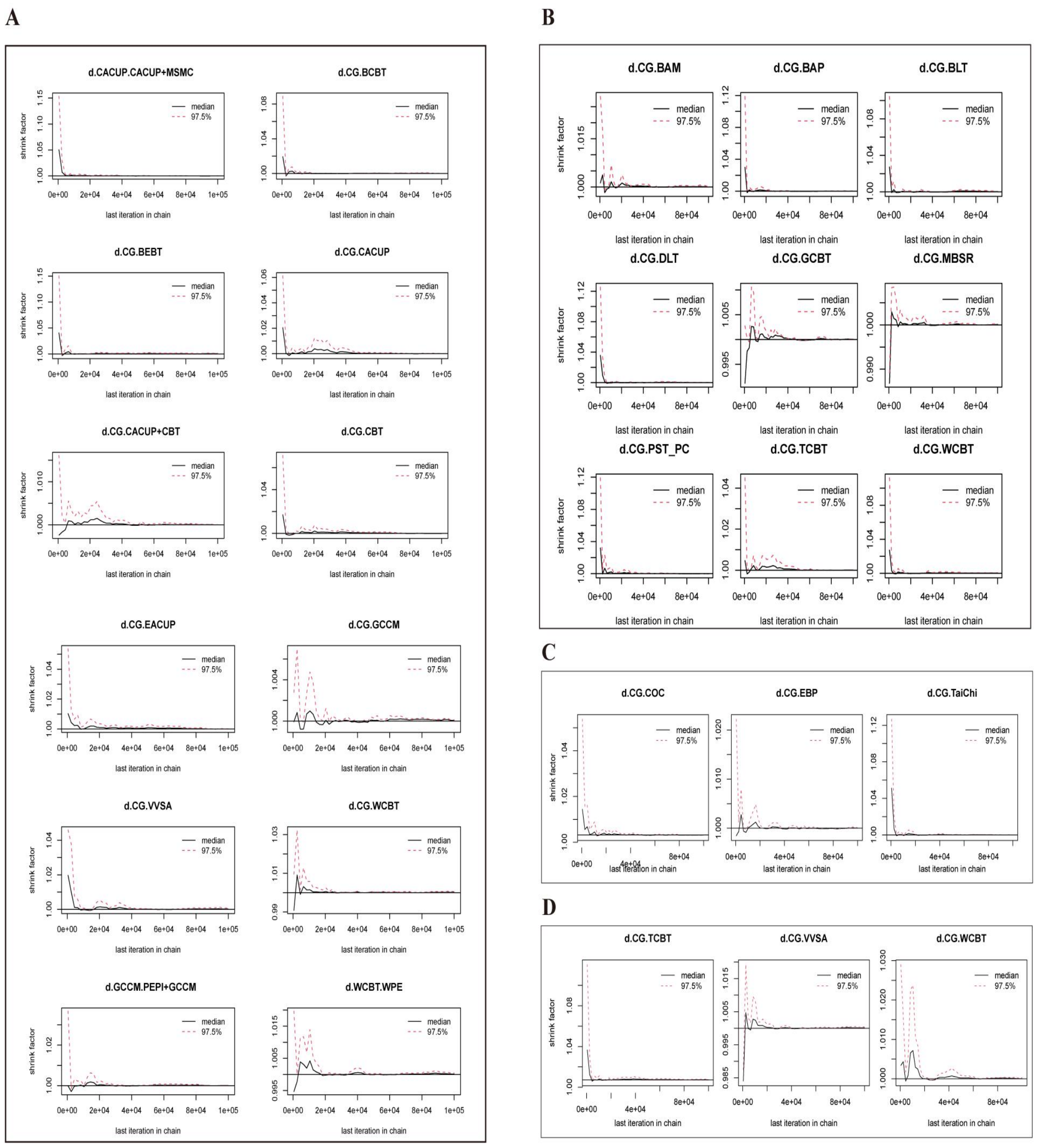

Supplementary Figure 3. Funnel plot for effect size and standard error of the included studies. (A) CES-D; (B) BDI; (C) PHQ-9; (D) K-6. Abbreviations: CES-D, Center for Epidemiologic Studies Depression Scale; BDI, Beck Depression Inventory Scale; PHQ-9, the 9-item Patient Health Questionnaire; K6, Kessler Screening Scale for Psychological Distress.

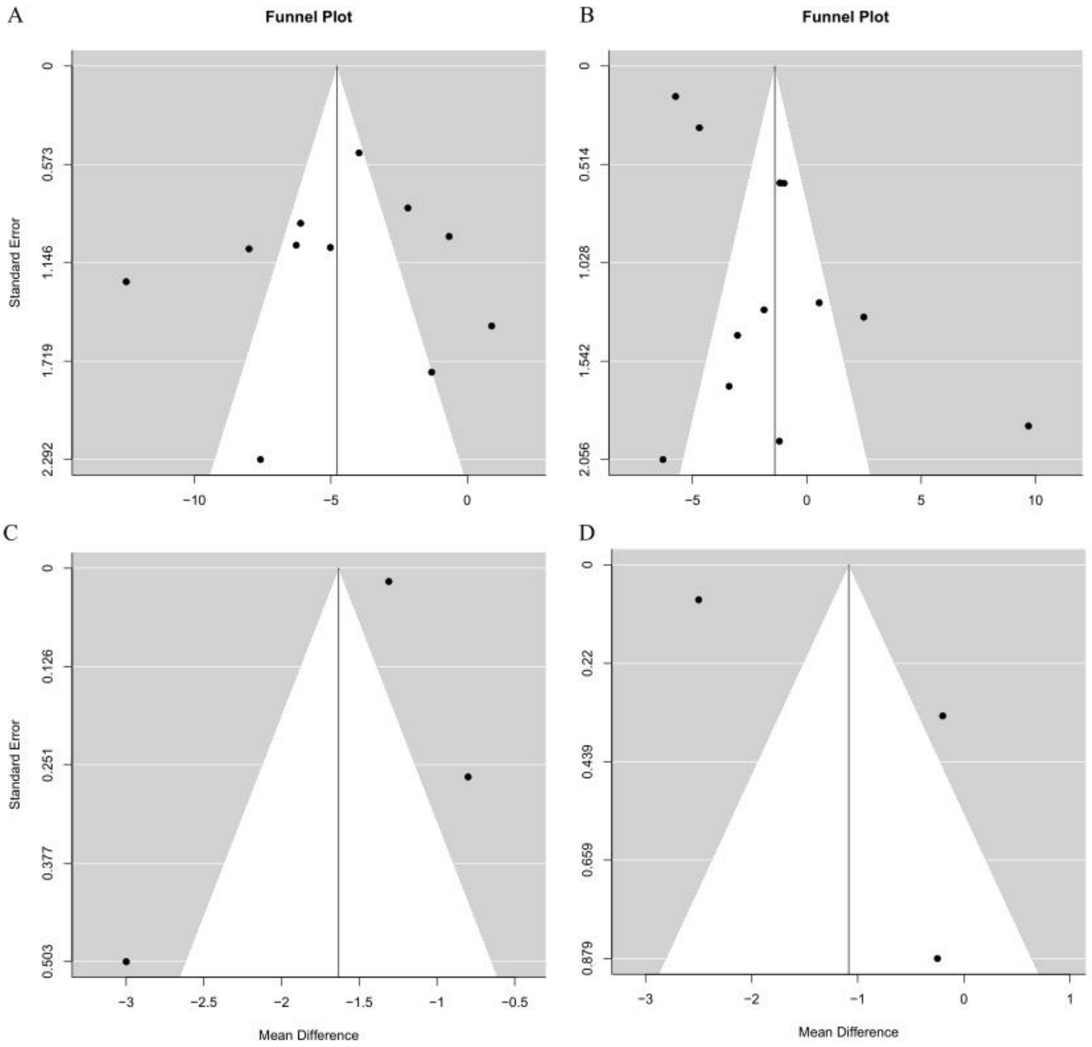

2. Supplementary table

Supplementary Table 1. Assessment of publication bias by Begg's and Egger's tests and model fit. Abbreviations: CES-D, Center for Epidemiologic Studies Depression Scale; BDI, Beck Depression Inventory Scale; PHQ-9, the 9-item Patient Health Questionnaire; K6, Kessler Screening Scale for Psychological Distress.

Outcome	P-value		Model fit	
	Egger's test	Begg's test	I^2	data points
BDI	0.213	0.947	7	18
CES	0.896	0.648	5	22
K6	0.277	1.000	17	6
PHQ	0.328	1.000	17	6
